# Surface-Collision Analysis of Microscale-Confined ^129^Xe in Pyrex Vapor Cells Based on Stem-Transport and Gradient Diffusion Dynamics

**DOI:** 10.3390/ma19050956

**Published:** 2026-03-01

**Authors:** Shangtao Jiang, Tengyue Wang, Xuyang Qiu, Heng Yuan

**Affiliations:** 1 School of Instrumentation and Optoelectronics Engineering, Beihang University, Beijing 100191, China; 2 Hangzhou Innovation Institute, Beihang University, Hangzhou 310052, China; 3 Hefei National Laboratory, Hefei 230088, China

**Keywords:** microfabricated vapor cells, cavity–stem geometry, structural coupling factor (SCF), transverse relaxation time *T*_2_, surface relaxation, gradient-induced dephasing

## Abstract

Surface collisions at Pyrex walls limit the spin coherence in nuclear magnetic resonance gyroscopes (NMRG) vapor cells, while the cavity–stem junction introduces geometry dependent exchange that perturbs the transverse spin relaxation time $T_{2}$ of ^129^Xe atoms. We combine $T_{2}$ measurements with Monte Carlo simulations of confined diffusion and surface collisions to decompose the relaxation of Xe atoms and derive a cavity–stem geometry correction for wall relaxation. A structural coupling factor (SCF) is introduced to compress stem length and aperture diameter into a dimensionless metric for diffusion-limited mixing, enabling prediction of the transverse relaxation rate versus geometry. Across eight simulated configurations, the model yields $R^{2}=0.982$ and agrees with experiments within 7–9%, comparable to the measurement uncertainty ($\pm 0.015\;s^{-1}$). Using the validated framework, geometry optimization reduces the relaxation rate from 0.225 to $0.131\;s^{-1}$ (a 41.8% improvement). This Pyrex surface-collisional analysis provides an in-situ, $T_{2}$-based route to compare effective surface depolarization across fabrication and surface-treatment protocols while accounting for cavity–stem coupling.

## 1. Introduction

Nuclear magnetic resonance gyroscopes (NMRGs) and alkali-metal–noble-gas comagnetometers require long-lived noble-gas coherence to achieve high stability and compact size in inertial sensing applications [1,2]. In these systems, the transverse relaxation time $T_{2}$ of ^129^Xe determines the linewidth of free-induction-decay (FID) measurement and coherent integration time, thereby imposing a central performance constraint in miniaturized vapor cells [3]. Diffusion-driven transverse relaxation in inhomogeneous magnetic fields is well described within the Bloch-Torrey and restricted-diffusion framework [4,5,6], where relaxation arises from the interplay of diffusion, magnetic-field gradients, and boundary interactions. As the cell dimensions shrink and the surface-to-volume ratio increases, wall-induced depolarization becomes an increasingly dominant limitation to coherence performance [7,8].

Microfabrication has enabled vapor cells with coupled cavity–stem geometries, where aperture diameter and stem length strongly influence diffusion pathways and wall-collision statistics [9]. Donley et al. demonstrated microfabricated noble-gas cells with preserved nuclear coherence [10], and Kitching highlighted geometry constraints in chip-scale atomic devices [11]. Wang et al. experimentally investigated stem-length effects on polarization-induced gradient relaxation in Xe comagnetometers [12]. Subsequent studies focused on performance improvement through gradient compensation, buffer-gas optimization, surface treatments, and signal-processing strategies [13,14,15,16]. Wu et al. analyzed surface-coating effects on Xe relaxation [17], and Xu et al. examined relaxation mechanisms in confined vapor cells [18]. Parallel theoretical efforts developed statistical and transport-based descriptions of confined spin dynamics and boundary-related relaxation [16,19,20]. Despite these advances, geometry-induced diffusion redistribution and intrinsic surface depolarization are often treated implicitly or remain coupled within geometry-specific simulations, limiting predictive comparison across stem–aperture configurations and hindering geometry-normalized assessment of inner-surface effects.

In contrast to prior studies that primarily focus on performance optimization or treat geometry-dependent effects implicitly within simulations, this work establishes an experimentally constrained and geometry-resolved description of wall-induced transverse relaxation. By explicitly linking cavity–stem structure to relaxation behavior through a compact structural descriptor, the proposed framework enables geometry-normalized comparison and provides a physically transparent basis for predictive design of miniature ^129^Xe vapor-cell systems.

## 2. Principle and Method

### 2.1. Structure-Weighted Diffusion and Boundary-Induced Relaxation

Transverse relaxation of diffusing ^129^Xe atoms in static field inhomogeneity is treated within the standard Bloch-Torrey/restricted-diffusion framework [4,5,21,22], with low-field gas scaling discussed by Cates et al. [6]. The gradient-induced contribution is written as

(1) $$\Gamma_{G}=\frac{\gamma^{2}}{2}\int_{-\infty }^{+\infty }\langle \delta B_{z}\left(0\right)\delta B_{z}\left(\tau \right)\rangle \;d\tau ,$$

where γ is the gyromagnetic ratio, $B_{z}$ is the magnetic field along the z-axis. For the linear magnetic field gradients, $\delta B_{z}\approx \;G\cdot \delta \;r$ [23], then

(2) $$\Gamma_{G}\approx \gamma^{2}\sum_{i}G_{i}^{2}\sigma_{i}^{2}\tau_{i},$$

where $\sigma_{i}^{2}$ and $\tau_{i}$ are set by restricted diffusion and the geometry of vapor cells. In cavity–aperture–stem cells, the aperture diameter *d* and stem length *h* redistribute diffusion trajectories through the cavity–stem exchange. A reduced two-region form is expressed by,

(3) $$\sigma_{z}^{2}=\left(1-P_{s}\right)\sigma_{z,c}^{2}+P_{s}\sigma_{z,s}^{2}+P_{s}\left(1-P_{s}\right){\left(\mu_{s}-\mu_{c}\right)}^{2},$$

The equation highlights that geometry enters through the stem occupancy $P_{s}\left(d,h\right)$, defined as the probability for atoms to reside in the stem region, which represents a structure-weighted sampling of magnetic-field inhomogeneity and internal boundaries. In later sections, this exchange effect is condensed into a structural coupling factor (SCF) for design-oriented comparison.

### 2.2. Polarization-Field Mapping and Gradient Metrics from COMSOL Simulations

The field inhomogeneity sampled by Xe atoms is generated by polarized Rb electrons. Following standard spin-exchange optical pumping (SEOP) descriptions [24,25,26], the coupled Bloch-Torrey equations for Rb and ^129^Xe polarization are solved in COMSOL Multiphysics 6.4 software to obtain steady-state $P_{\mathrm{Rb}}\left(r\right)$ and $P_{\mathrm{Xe}}\left(r\right)$ [27]:

(4) $$\begin{array}{cc} \frac{\partial P_{\mathrm{Rb}}}{\partial t} & =\gamma_{e}P_{\mathrm{Rb}}\times B+D_{\mathrm{Rb}}\nabla^{2}P_{\mathrm{Rb}}+R_{\mathrm{op}}\left(P_{0}\hat{z}-P_{\mathrm{Rb}}\right)-R_{1,\mathrm{Rb}}P_{\mathrm{Rb}}, \end{array}$$

(5) $$\begin{array}{cc} \frac{\partial P_{\mathrm{Xe}}}{\partial t} & =\gamma_{n}P_{\mathrm{Xe}}\times B+D_{\mathrm{Xe}}\nabla^{2}P_{\mathrm{Xe}}+R_{\mathrm{se}}\left(P_{\mathrm{Rb}}-P_{\mathrm{Xe}}\right)-R_{1,\mathrm{Xe}}P_{\mathrm{Xe}}. \end{array}$$

where $P_{\mathrm{Rb}}$ and $P_{\mathrm{Xe}}$ are the spin polarization of Rb and Xe atoms, *B* is the magnetic field, *D* is the diffusion coefficient of atoms, $R_{\mathrm{op}}$ is the optical pumping rate, and *R* is the relaxation rate. The Rb polarization is converted to the effective field experienced by Xe via the Fermi-contact interaction,

(6) $$B_{\mathrm{eff}}\left(r\right)=\frac{1}{3}\mu_{0}\mu_{B}g_{s}\kappa_{0}n_{\mathrm{Rb}}P_{\mathrm{Rb}}\left(r\right),$$

which provides $\delta B_{z}\left(r\right)$ and its gradients as the input for evaluating $\Gamma_{G}$.

The nuclear polarization field $P_{\mathrm{Xe}}\left(r\right)$ is retained to support controlled model reduction for stemmed cells. Specifically, COMSOL-derived polarization/field metrics are used later to justify the separation $\Gamma_{\mathrm{total}}\left(h,d\right)=\Gamma_{\mathrm{wall}}\left(h,d\right)+\Gamma_{G}\left(d\right)$ (Equation (10)) only in the experimentally relevant range where the effective gradient sampling shows weak dependence on *h*.

### 2.3. Monte Carlo Modeling of FID Signals and Effective Surface Parameterization

**Figure 1:**
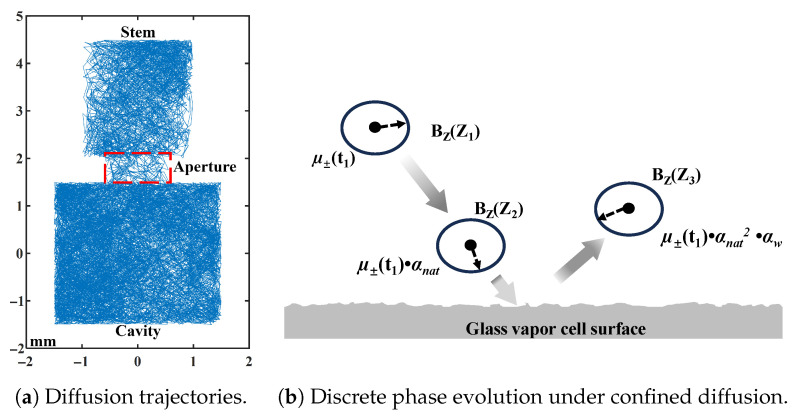
Monte Carlo modeling of confined diffusion and transverse phase evolution. (**a**) Random-walk trajectories of atoms confined within the cavity–aperture–stem geometry, illustrating geometry-dependent spatial exploration. (**b**) Time-discretized accumulation of transverse phase ϕ(t) used to compute ensemble magnetization decay, following diffusion-mediated spin-relaxation theory.

Relaxation in the full cavity–aperture–stem geometry is evaluated by a 3D Monte Carlo random walk [4,21]. The diffusive step is $L_{d}=\sqrt{6D\Delta t}$ and the phase evolves as $\phi \left(t+\Delta t\right)=\phi \left(t\right)+\gamma \;\delta B_{z}\left(r\left(t\right)\right)\Delta t$. Representative trajectories illustrate geometry-dependent exchange and boundary sampling, and the discrete phase accumulation used to compute $M_{⊥}\left(t\right)=\langle e^{i\phi (t)}\rangle$ is shown in Figure 1. The total decay rate Γ is extracted by exponential fitting of $|M_{⊥}\left(t\right)|$.

We decompose the total relaxation as

(7) $$\Gamma =\Gamma_{G}+\Gamma_{\mathrm{wall}},\;\Gamma_{\mathrm{wall}}≃\lambda_{\mathrm{MC}}\left[-\ln(\alpha_{\mathrm{eff}})\right],\;\alpha_{\mathrm{eff}}=\exp\;\left(-\frac{1}{\lambda_{\mathrm{MC}}T_{2}^{\exp}}\right).$$

where $\Gamma_{\mathrm{wall}}$ is the wall collisions of atoms, $\alpha_{\mathrm{eff}}$ represents the effective spin-preservation probability per wall collision and $\lambda_{\mathrm{MC}}$ denotes Monte Carlo extracted wall-collision frequency.

## 3. Experimental Setup and Procedure

### 3.1. Experimental Setup

**Figure 2:**
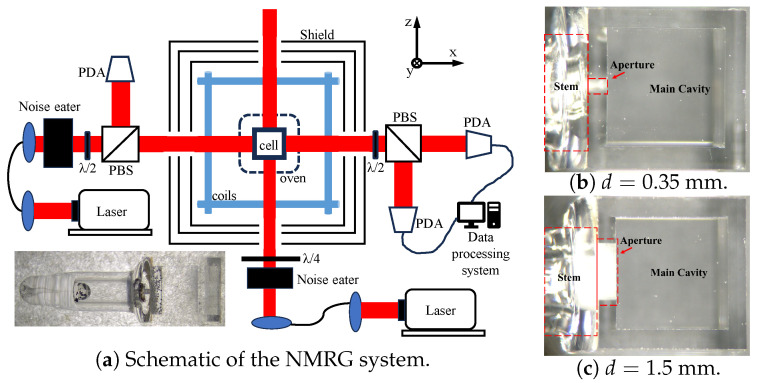
Experimental setup and the stem-aperture-cavity structure. (**a**) Schematic of the NMRG system. (**b**,**c**) Pictures of stemmed vapor cells with aperture diameters of 0.35mm and 1.5mm, respectively.

Figure 2a shows the experimental configuration. Two DBR lasers were used as pump and probe sources, tuned to the Rb D_1_ and D_2_ transitions, respectively. The pump beam was circularly polarized and expanded to a 2.5 mm Gaussian profile with stabilized power of 4 mW. Optical pumping polarized ^87^Rb atoms, which transferred polarization to Xe nuclei via spin exchange [28]. The probe beam was linearly polarized and slightly detuned to detect transverse spin precession through Faraday rotation [29]. A lock-in amplifier (Zurich Instruments, Zürich, Switzerland, HF2LI) performed phase-sensitive detection. Gradient coils provided magnetic-field-gradient generation and compensation. A four-layer magnetic shield reduced the environmental magnetic interference [30].

The cubic cavity has an inner side length of 3 mm, corresponding to a volume of approximately 27 mm^3^, while the stem contributes less than 5% additional volume in the vapor cell. The cell contains ^87^Rb, 2 Torr ^129^Xe, 8 Torr ^131^Xe, and 300 Torr N_2_, and operates at the temperature of 393 K. Cells with aperture diameters of 0.35 mm and 1.5 mm were fabricated, and the initial 2 mm stem was progressively shortened to tune the structural coupling factor Λ(h,d) (Equation (13)). Figure 2b,c shows the vapor cells fabricated for experimental validation. The geometry was systematically tuned by progressively shortening the stem while preserving the integrity of the cubic sensing cavity. Although laser-based trimming may introduce localized thermal stress or surface roughness, the processed region lies outside the active sensing volume and does not participate in optical pumping or detection. No systematic change in the baseline transverse relaxation rate beyond measurement repeatability was observed after processing.

### 3.2. Measurement of Free Induction Decay Signals

**Figure 3:**
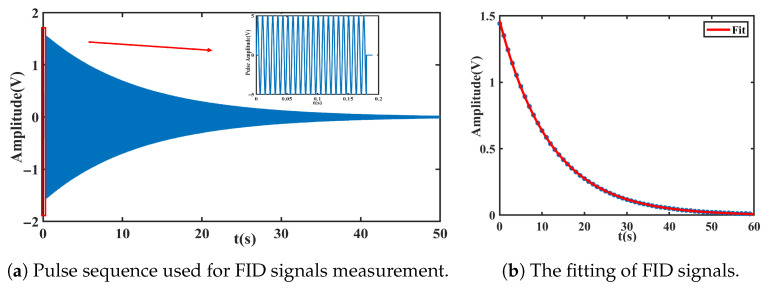
The measurement of Free-induction-decay (FID) signals. (**a**) Experimental pulse sequence used to initiate transverse spin precession. (**b**) Typical FID signal showing exponential transverse relaxation behavior.

As illustrated in the inset of Figure 3a, a π/2 pulse tips the Xe nuclear magnetization into the transverse plane. After the pulse is turned off, the transverse magnetization undergoes free precession at the Larmor frequency while decaying exponentially in time. The underlying principles follow standard spin-echo theory [31,32] as described in classical magnetic resonance literature [33]. The decay envelope is fitted to extract the transverse relaxation time $T_{2}$, and repeated measurements are performed to evaluate the associated statistical uncertainty. A representative single-shot free-induction-decay signal is shown in Figure 3b.

## 4. Results and Discussion

**Figure 4:**
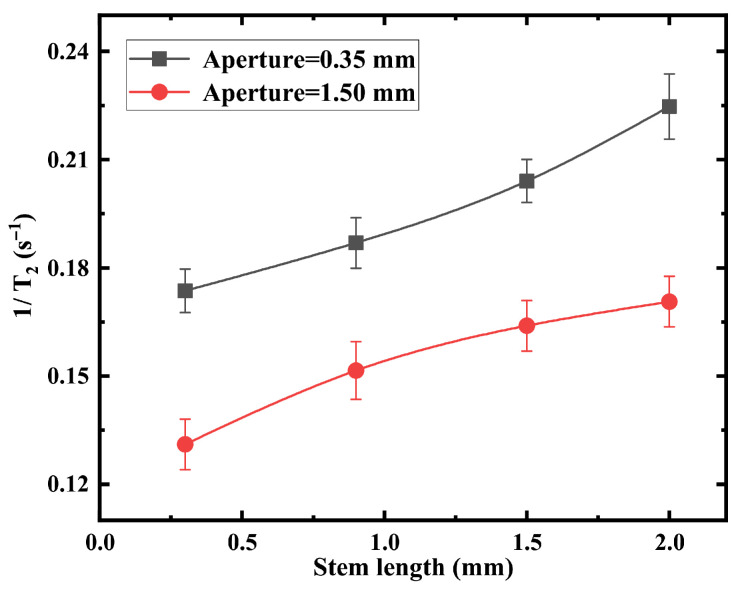
Experimental relaxation rate as a function of stem length under different vapor cell apertures.

**Figure 5:**
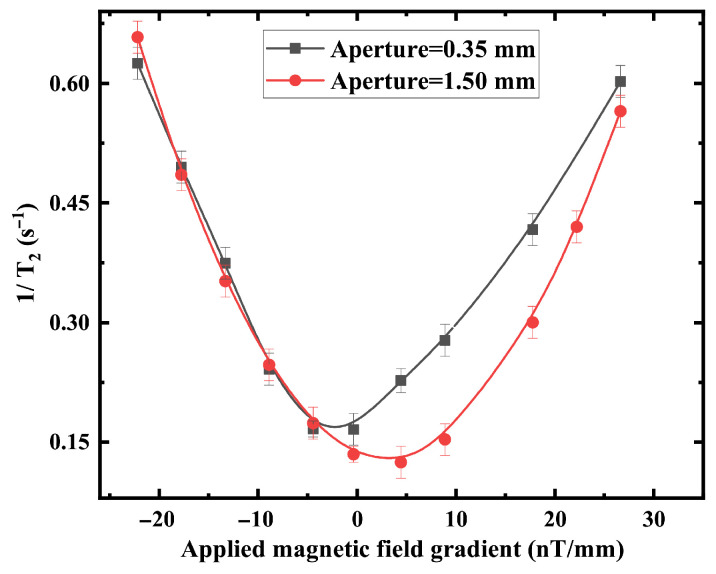
Experimental gradient-dependent relaxation for a stem length h=0.3mm. Measured relaxation rate versus applied magnetic field gradient. The solid lines represent a quadratic fit for each aperture.

Figure 4 shows the compensated transverse relaxation rate as a function of the length of the vapor cell stem. Shortening the stem consistently reduces the relaxation rate for both aperture diameters. The larger aperture configuration achieves systematically lower relaxation, confirming the simulation trend. Reducing h and enlarging d can suppress the transverse relaxation rate from $0.225\;s^{-1}$ to $0.131\;s^{-1}$, corresponding to a reduction of 41.8%. For the stem-length dependence (Figure 5), comparison between the repeated measured relaxation rates gives mean absolute percentage errors of 4–5%.

Figure 5 confirms the quadratic dependence of the transverse relaxation rate on magnetic-field gradient magnitude, consistent with diffusion theory in confined geometries. The curvature of the quadratic dependence decreases for geometries with larger apertures or shorter stems, indicating reduced sensitivity to gradient-induced dephasing. For the gradient-dependent measurements (Figure 4), quadratic fitting of $1/T_{2}$ versus applied gradient yields $R^{2}=0.997$ for d=0.35 mm and $R^{2}=0.994$ for d=1.50 mm, with maximum relative deviations below 5%. The repeated experimental uncertainties (error bars) of $1/T_{2}$ are predominantly within $\pm 0.02\;s^{-1}$, with a few data points showing $\pm 0.01\;s^{-1}$ or $\pm 0.015\;s^{-1}$. Considering that $1/T_{2}$ mainly ranges from 0.2 to $0.6\;s^{-1}$, the corresponding relative uncertainties are approximately 3–10% (typically around 5%), indicating influence of the reproducibility and data dispersion on the overall trend.

These results demonstrate that geometry modifies the effective sampling of magnetic-field gradients by diffusing atoms, rather than merely shifting the baseline relaxation rate. The observed trend cannot be explained solely by cavity volume change, indicating a geometry-mediated diffusion effect. To distinguish gradient-induced relaxation from wall-collision contributions, we extracted effective polarization-gradient metrics from COMSOL point clouds [34].

**Figure 6:**
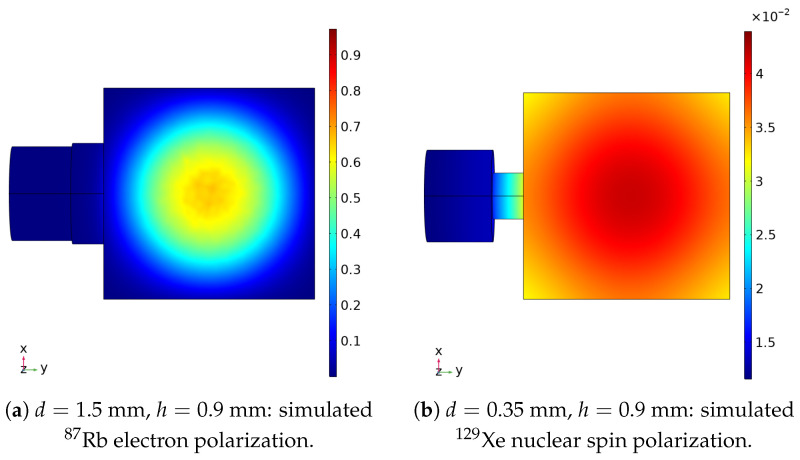
Steady-state polarization distributions simulated by COMSOL. (**a**) Spatial distribution of the normalized ^87^Rb electron polarization, which can be converted into the effective magnetic field experienced by ^129^Xe through the Feimi contact interaction. (**b**) Spatial distribution of the normalized ^129^Xe nuclear spin polarization, reflecting the dynamic balance between hyperpolarization, diffusion, and relaxation. Both simulations reveal a clear contrast between the cavity and stem regions, governed by the coupled effects of optical pumping, diffusive motions, and spin relaxation.

By solving Equation (11), the distribution of Rb electron polarization can be simulated. The parameters can be referred to our former work [35], as is shown in Figure 6. The COMSOL steady-state electron polarization $P_{\mathrm{Rb}}\left(r\right)$ is used to derive an effective field

(8) $$B_{\mathrm{eff}}\left(r\right)=C\;P_{\mathrm{Rb}}\left(r\right),\;G_{\mathrm{rms}}=\sqrt{{\langle |\nabla B_{\mathrm{eff},z}{\left(r\right)|}^{2}\rangle }_{V}}.$$

Gradients are computed from exported COMSOL point clouds using local linear reconstruction on irregular nodes, as is shown in Table 1. For geometry comparison, we use the cubic scaling normalization

(9) $$\Gamma_{G}=\frac{\gamma^{2}L^{4}}{120D}\;G_{\mathrm{rms}}^{2},$$

Within the investigated range, gradient metrics vary mainly with *d* and only weakly with *h* (Appendix A). We therefore write

(10) $$\Gamma_{\mathrm{total}}\left(h,d\right)=\Gamma_{\mathrm{wall}}\left(h,d\right)+\Gamma_{G}\left(d\right),$$

and approximate

(11) $$\Gamma_{G}\left(d\right)\approx G_{0}\left(d\right),$$

with

$$G_{0}\left(0.35\;\mathrm{mm}\right)=0.020\;s^{-1},\;G_{0}\left(1.50\;\mathrm{mm}\right)=0.015\;s^{-1}.$$

To capture the stem-length dependence, we introduce (Appendix B)

(12) $$\Gamma_{\mathrm{wall}}=\Gamma_{0}+\beta \frac{h}{h+\alpha d^{2}},$$

**Figure 7:**
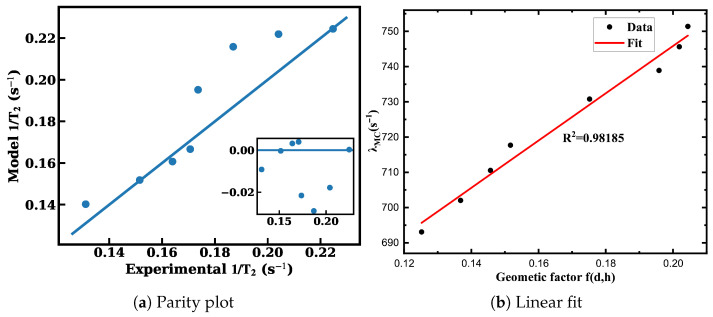
Validation of the geometry-resolved wall-relaxation model. (**a**) Monte Carlo extracted wall-collision frequency $\lambda_{\mathrm{MC}}$ plotted against the proposed geometric factor $f\left(d,h\right)=0.118+0.095\;h/\left(h+1.62d^{2}\right)$. The solid line represents the linear fit, and the coefficient of determination $R^{2}$ is shown in the panel. (**b**) Global comparison between model-predicted and experimentally measured transverse relaxation rates for eight geometries. The solid line indicates the unity-slope reference, and the inset shows residuals $\Gamma_{\exp}-\Gamma_{\mathrm{model}}$, demonstrating the absence of systematic deviation.

As is shown in Figure 7a, simultaneous least-squares fitting to eight experimental data points yields

(13) $$\Gamma_{0}=0.118\;s^{-1},\;\beta =0.095,\;\alpha =1.62,\;\Lambda \left(h,d\right)=\frac{h}{h+\alpha d^{2}}.$$

The model gives $R^{2}=0.973$ with maximum relative deviation below 6.5%. The SCF form $\Lambda \left(h,d\right)=h/\left(h+\alpha d^{2}\right)$ follows from diffusion conductance through a circular aperture (G∼DA/h with $A\propto d^{2}$) together with a two-compartment exchange model in which independent decorrelation channels add in rate; see Appendix B for the full derivation and definitions. The coefficient 1.62 is fixed by Monte Carlo exchange statistics under the present geometry and operating conditions.

To validate the proposed geometric wall-collision factor

(14) $$f\left(d,h\right)=0.118+0.095\frac{h}{h+1.62d^{2}},$$

As is shown in Figure 7b, a linear regression between $\lambda_{\mathrm{MC}}$ and f(d,h) yields

(15) $$\lambda_{\mathrm{MC}}=670.526\;f\left(d,h\right)+611.714,\;R^{2}=0.98185.$$

Under the condition of dominated wall-collisions relaxation,

(16) $$\frac{1}{T_{2,\mathrm{wall}}}\approx \lambda_{\mathrm{MC}}\left[-\ln(\alpha_{\mathrm{eff}})\right],\;\alpha_{\mathrm{eff}}=\exp\left(-\frac{1}{\lambda_{\mathrm{MC}}T_{2}^{\exp}}\right).$$

The values in Table 2 are averaged over all experimental geometries yields

(17) $$\alpha_{\mathrm{eff}}=0.999758,\;-\ln\left(\alpha_{\mathrm{eff}}\right)=2.42\times 10^{-4}.$$

After extracting $\alpha_{\mathrm{eff}}$, the Monte Carlo simulation of the FID process was carried out under the same diffusion and geometry conditions. Substituting the linear relation of $\lambda_{\mathrm{MC}}$ gives a fully analytic relaxation model.

(18) $$T_{2,\mathrm{wall}}\left(d,h\right)=\frac{1}{\left(675.239f(d,h)+611.427\right)\left[-\ln(\alpha_{\mathrm{eff}})\right]}.$$

The simulated FID decay is then directly compared with the experiment.

To quantitatively evaluate the agreement between experiment, Monte Carlo simulation combining analytical model, the relaxation trends were compared under two structural variations: (i) stem length *h* and (ii) applied magnetic-field gradient.The relative deviation was calculated as

(19) $$\delta =\frac{\left|{\left(1/T_{2}\right)}_{\exp}-{\left(1/T_{2}\right)}_{\mathrm{sim}}\right|}{{\left(1/T_{2}\right)}_{\mathrm{sim}}}\times 100\%.$$

**Figure 8:**
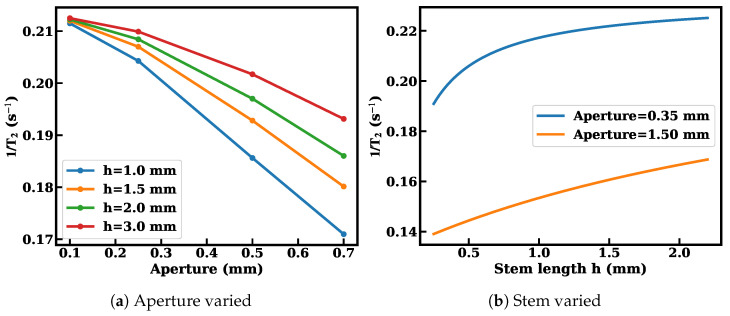
Geometry-weighted wall relaxation governs the transverse relaxation dependence on aperture diameter and stem length.

First, the relaxation rate exhibits a consistent monotonic dependence on stem length across both experimental (Figure 4) and theoretical results (Figure 8b). Increasing *h* leads to an increase in $\Gamma =1/T_{2}$, reflecting enhanced wall-collision probability and extended diffusion residence in the stem region. This trend is reproduced by the theoretical model and Monte Carlo simulations, indicating that the geometry-weighted wall contribution is captured correctly at the leading order.The relative deviation between experiment and theory for the eight data points remains within approximately 6∼9%.

**Figure 9:**
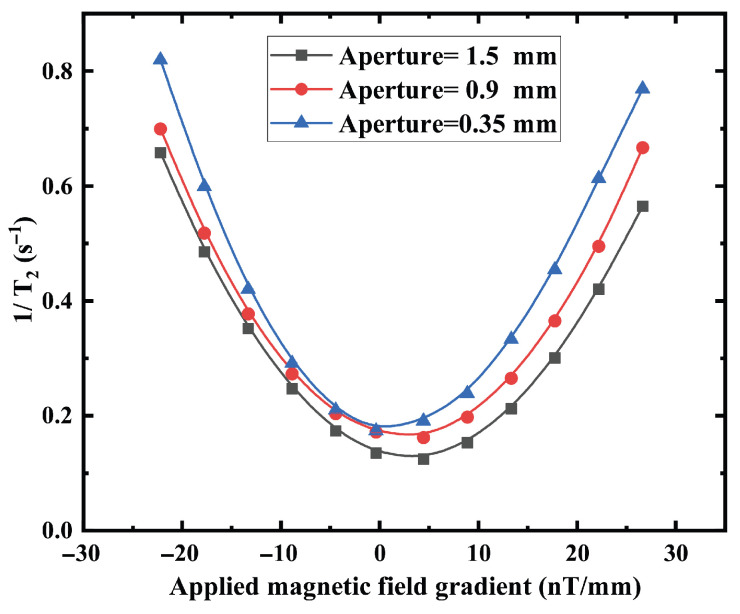
Simulated relaxation rate as a function of the applied magnetic-field gradient.

Second, under applied magnetic-field gradients, the relaxation rate follows a quadratic dependence, consistent with diffusion-mediated dephasing theory. To quantitatively evaluate the agreement between theoretical trend (Figure 9) and experimental data (Figure 5). Across the full magnetic field range, the relative deviations are predominantly below 5%, with an average relative deviation of approximately 3–4%, and a maximum deviation below 10% occurring at low-field conditions.

The semi-empirical linear magnetic field gradient assumptions and simplified wall-collision boundary conditions introduce theoretical uncertainties in the Monte Carlo model. In addition, laser-based stem shortening, surface roughness and stress, or contamination may contribute to experimental uncertainty. Together, these factors account for the small but measurable discrepancies between simulation and experiment. A baseline offset exists between model prediction and experiment, and small differences in quadratic curvature (concavity) appear under gradient scans. Quantitatively, for the d=1.50 mm aperture, the root-mean-square error (RMSE) is approximately $0.005\;s^{-1}$, indicating excellent agreement. In contrast, for the d=0.35 mm aperture, the RMSE increases to approximately $0.02\;s^{-1}$.

This suggests that under smaller aperture conditions, either the wall-relaxation contribution or the effective gradient correction term may be slightly underestimated. Alternatively, additional surface-related depolarization mechanisms may contribute in the experiment but are not fully captured in the present model. The overall deviation magnitude is approximately $0.015\;s^{-1}$ while the measured relaxation range is 0.15–$0.22\;s^{-1}$, corresponding to a relative error of approximately 6–9%. Considering that the experimental uncertainty of $1/T_{2}$ is on the order of 3–10%, the observed deviations fall within or close to the experimental error bounds. This confirms quantitative consistency within experimental uncertainty.

The model assumes linear magnetic-field gradients and idealized wall-collision boundary conditions in the Monte Carlo simulations. Each FID configuration was measured five times; the error bars reflect the standard deviation from exponential fitting. Within these uncertainties, the trends are reproducible, and validation across 20 geometries (Table 1) provides an internal consistency check on the extracted wall parameter and gradient contribution, respectively.

## 5. Conclusions

This work combines systematic $T_{2}$ measurements with Monte Carlo simulations of surface-collision statistics and introduces SCF to describe diffusive exchange between the sensing cavity and the stem. Based on this framework, we establish a relaxation model that relates the measured transverse relaxation rate to stem length and aperture size. Fitting is performed on eight experimentally tested configurations with a coefficient of determination $R^{2}=0.982$, and the framework is cross-checked against 20 simulated geometries. Repeated FID measurements yield uncertainties (error bars) within $\pm 0.015\;s^{-1}$. The model-predicted relaxation rates differ from the experimental values by approximately 6–9%, remaining within or near the experimental uncertainty range. Guided by the model, geometry optimization reduces the relaxation rate from $0.225\;s^{-1}$ to $0.131\;s^{-1}$, corresponding to a 41.8% improvement in coherence performance. From a materials perspective, this Pyrex surface-collisional analysis supports in-situ, $T_{2}$-based comparison of effective surface depolarization across fabrication routes and surface treatments, while explicitly accounting for the cavity–stem junction that can bias the apparent relaxation. This offers a practical tool for microscale vapor-cell design and evaluation: once cavity–stem exchange and gradient–diffusion effects are accounted for, $T_{2}$ can be used to compare effective surface depolarization across fabrication, cleaning, and coating protocols. Future work will refine surface-loss models, correlate the effective surface term with independent surface metrology, and extend the approach to other cell architectures and materials.

## Figures and Tables

**Table 1:** Geometry-resolved metrics for gradient-induced and wall-induced transverse relaxation. For each geometry (d,h), COMSOL-derived gradient relaxation parameters (left) and Monte Carlo wall-collision-based parameters (right) are listed for direct comparison.

	COMSOL: Gradient-Induced				Monte Carlo: Wall-Collision		
(d,h)(mm)	Λ	$G_{\mathrm{rms}}$	$\Gamma_{G}$	$T_{2,G}$	$f_{\mathrm{geom}}$	$\lambda_{\mathrm{pred}}$	$T_{2,\mathrm{theory}}$
(0.35, 0.3)	0.603	21.100	0.0312	32.1	0.196	743.664	5.558
(0.35, 0.9)	0.825	21.060	0.0310	32.2	0.175	729.713	5.664
(0.35, 1.5)	0.885	20.300	0.0288	34.7	0.210	753.167	5.488
(0.35, 2.0)	0.907	19.610	0.0269	37.2	0.208	752.171	5.495
(1.50, 0.3)	0.074	20.210	0.0286	35.0	0.186	736.762	5.610
(1.50, 0.9)	0.216	18.760	0.0246	40.6	0.212	754.908	5.475
(1.50, 1.5)	0.357	18.190	0.0232	43.2	0.212	754.737	5.477
(1.50, 2.0)	0.471	17.400	0.0212	47.2	0.211	754.230	5.480
(0.10, 3.0)	0.995	12.860	0.0116	86.4	0.137	703.808	5.873
(0.10, 2.0)	0.992	17.280	0.0209	47.9	0.146	709.807	5.823
(0.10, 1.0)	0.984	21.480	0.0323	31.0	0.152	713.832	5.790
(0.25, 3.0)	0.967	12.580	0.0111	90.2	0.202	747.758	5.528
(0.25, 2.0)	0.952	16.200	0.0184	54.4	0.204	749.462	5.515
(0.25, 1.0)	0.910	20.280	0.0288	34.8	0.125	695.983	5.939
(0.50, 3.0)	0.857	16.080	0.0181	55.3	0.186	737.016	5.607
(0.50, 2.0)	0.800	17.050	0.0204	49.1	0.193	741.823	5.571
(0.50, 1.0)	0.667	21.290	0.0317	31.5	0.205	749.846	5.512
(0.70, 3.0)	0.756	14.150	0.0140	71.3	0.197	744.450	5.552
(0.70, 2.0)	0.668	18.920	0.0251	39.9	0.202	747.623	5.529
(0.70, 1.0)	0.507	20.100	0.0283	35.4	0.171	726.857	5.687

**Table 2:** Extracted $\alpha_{\mathrm{eff}}$ values based on Monte Carlo wall-collision frequencies.

Data	1	2	3	4	5	6	7	8
$\alpha_{\mathrm{eff}}$	0.999764	0.999747	0.999726	0.999701	0.999811	0.999784	0.999769	0.999762
Mean ± SD	$0.999758\pm (3.3\times 10^{-5}$)							

## Data Availability

The original contributions presented in this study are included in the article. Further inquiries can be directed to the corresponding author.

## Appendix A. Sensitivity Analysis of Gradient Metrics to *h* and *d*

**Table A1:** Effective-gradient metrics extracted from COMSOL polarization point clouds. $G_{\mathrm{rms}}^{\left(P\right)}$ denotes the RMS magnitude of the local polarization gradient reconstructed by k-nearest-neighbor linear fitting on irregular nodes, which is proportional to $G_{\mathrm{rms}}$ used in Equation (9) via $B_{\mathrm{eff}}=CP_{e}$. $G_{99}$ is the 99th percentile of $|\nabla P_{e}|$, and $f_{\mathrm{hot}}$ is the fraction of points satisfying $|\nabla P_{e}|>2\;\mathrm{median}(|\nabla P_{e}\left|\right)$.

Set A						Set B					
d (mm)	h (mm)	r=h/d	$G_{\mathrm{rms}}^{\left(P\right)}$ (1/mm)	$G_{99}$ (1/mm)	$f_{\mathrm{hot}}$	d (mm)	h (mm)	r=h/d	$G_{\mathrm{rms}}^{\left(P\right)}$ (1/mm)	$G_{99}$ (1/mm)	$f_{\mathrm{hot}}$
0.10	1.0	10.000	0.275	1.112	0.264	0.50	1.0	2.000	0.273	0.836	0.285
0.10	2.0	20.000	0.221	0.891	0.264	0.50	2.0	4.000	0.218	0.704	0.270
0.10	3.0	30.000	0.165	0.654	0.273	0.50	3.0	6.000	0.206	0.733	0.256
0.25	1.0	4.000	0.260	0.824	0.275	0.70	1.0	1.429	0.257	0.816	0.275
0.25	2.0	8.000	0.208	0.704	0.247	0.70	2.0	2.857	0.242	0.736	0.294
0.25	3.0	12.000	0.161	0.590	0.266	0.70	3.0	4.286	0.181	0.643	0.252
0.35	0.3	0.857	0.270	0.837	0.275	1.50	0.3	0.200	0.259	0.720	0.318
0.35	0.9	2.571	0.270	0.827	0.279	1.50	0.9	0.600	0.240	0.792	0.268
0.35	1.5	4.286	0.260	0.811	0.260	1.50	1.5	1.000	0.233	0.773	0.264
0.35	2.0	5.714	0.251	0.787	0.266	1.50	2.0	1.333	0.223	0.772	0.273

To quantify the relative influence of stem length *h* and aperture diameter *d* on the effective gradient metric, we performed a global linear regression. This sensitivity comparison is motivated by restricted-diffusion theory in bounded regions, which links geometry-dependent sampling of inhomogeneous fields to relaxation and dephasing behavior [21].

(A1) $$G_{\mathrm{rms}}^{\left(P\right)}\approx a\;h+b\;d+c.$$

The least-squares fit yields

(A2) $$\frac{\partial G_{\mathrm{rms}}^{\left(P\right)}}{\partial h}\approx -3.7\times 10^{-2}\;{\mathrm{mm}}^{-2},\;\frac{\partial G_{\mathrm{rms}}^{\left(P\right)}}{\partial d}\approx -9.9\times 10^{-3}\;{\mathrm{mm}}^{-2}.$$

Within the experimentally relevant apertures (d=0.35 mm and 1.5 mm), varying *h* over the investigated range modifies $G_{\mathrm{rms}}^{\left(P\right)}$ by approximately 7–14%. A similar weak dependence on *h* is observed in the high-gradient tail metric $G_{99}$ (the 99th percentile of $|\nabla P_{e}|$), which varies by less than ∼10% within each fixed-*d* series. Moreover, the hot-region fraction

(A3) $$f_{\mathrm{hot}}=\mathrm{frac}\;\left(|\nabla P_{e}|>2\;\mathrm{median}(|\nabla P_{e}\left|\right)\right)$$

remains confined to the narrow range ∼0.26–0.32 across all tested *h* values, indicating that the spatial extent of strong-gradient regions is only weakly affected by stem length.

In contrast, changing the aperture diameter *d* produces substantially larger differences in the high-gradient structure, with $G_{99}$ varying by up to ∼55% across the full diameter range. These comparisons demonstrate that, in the experimentally relevant regime, the gradient-related contribution is significantly more sensitive to *d* than to *h*, not only in the global RMS metric but also in the extreme-gradient tail and hotspot fraction, as summarized in Table A1.

Accordingly, we adopt the approximation

(A4) $$\Gamma_{G}\left(h,d\right)\approx \Gamma_{G}\left(d\right)$$

within this validated parameter range, while retaining the full geometry dependence outside it to avoid extrapolation.

## Appendix B. Derivation of the Structural Coupling Factor (SCF): Transport-Limited Cavity–Stem Exchange

We model the vapor cell as two well-mixed compartments (main cavity volume $V_{c}$ and stem volume $V_{s}$) connected by a short channel of length *h* and a circular opening of diameter *d*. Transport theory gives the diffusion-limited conductance through the connection as

(A5) $$G∼\frac{DA}{h},$$

where *D* is the diffusion coefficient and *A* is the opening area [36,37]. For a circular opening, $A\propto d^{2}$.

In a two-compartment mass-balance description, the inter-compartment exchange rate constant scales with the conductance and the compartment volumes,

(A6) $$k_{\mathrm{ex}}\propto G\left(\frac{1}{V_{c}}+\frac{1}{V_{s}}\right),$$

so that combining Equations (A5) and (A6) yields the geometry scaling

(A7) $$k_{\mathrm{ex}}\propto \frac{D\;d^{2}}{V_{\mathrm{eff}}\;h},\;V_{\mathrm{eff}}^{-1}\equiv \left(\frac{1}{V_{c}}+\frac{1}{V_{s}}\right),$$

i.e., the $d^{2}$ dependence follows directly from the area scaling of diffusion-limited conductance, while the 1/h dependence follows from the diffusive resistance of the connecting channel.

In the motional-narrowing description of gradient-induced relaxation, the relevant field-correlation time is governed by the slowest mixing mode [5,6]. When a geometry-independent baseline randomization process with rate $k_{0}$ acts in parallel with inter-compartment exchange, the total decorrelation rate is

(A8) $$k_{\mathrm{tot}}=k_{0}+k_{\mathrm{ex}},\;\tau_{c}=\frac{1}{k_{0}+k_{\mathrm{ex}}}.$$

Defining a bounded, dimensionless structural coupling factor as the normalized correlation time,

(A9) $$\Lambda \equiv \frac{\tau_{c}}{\tau_{0}}=\frac{1}{1+k_{\mathrm{ex}}/k_{0}},\;\tau_{0}\equiv \frac{1}{k_{0}},$$

and substituting Equation (A7) gives

(A10) $$\Lambda \left(h,d\right)=\frac{1}{1+\alpha d^{2}/h}=\frac{h}{h+\alpha d^{2}},$$

where α collects prefactors (including *D*, $V_{\mathrm{eff}}$, and $\tau_{0}$) that are fixed for a given operating condition. Therefore, the functional dependence on *h* and $d^{2}$ is dictated by transport scaling and rate addition; only the single prefactor α is condition-dependent (and can be fixed by simulation or by a single fit for the given geometry/condition).
